# Controlling the uncontrollable: Quantum control of open-system dynamics

**DOI:** 10.1126/sciadv.add0828

**Published:** 2022-11-02

**Authors:** Shimshon Kallush, Roie Dann, Ronnie Kosloff

**Affiliations:** ^1^ Sciences Department, Holon Academic Institute of Technology, 52 Golomb Street, Holon 58102, Israel.; ^2^ The Institute of Chemistry, The Hebrew University of Jerusalem, Jerusalem 9190401, Israel.

## Abstract

Control of open quantum systems is essential for the realization of contemporary quantum science and technology. We demonstrate such control using a thermodynamically consistent framework, taking into account the fact that the drive can modify the system’s interaction with the environment. Such an effect is incorporated within the dynamical equation, leading to control-dependent dissipation. This relation serves as the key element for open-system control. The control paradigm is displayed by analyzing entropy-changing state-to-state transformations, such as heating and cooling. The difficult task of controlling quantum gates is achieved for nonunitary reset maps with complete memory loss. In addition, we identify a mechanism for controlling unitary gates by actively removing entropy from the system to the environment. We demonstrate a universal set of single- and double-qubit unitary gates under dissipation.

## Teaser

A set of universal quantum gates under open-system conditions is generated by a thermodynamically consistent control method.

## INTRODUCTION

Quantum control addresses the task of driving the state of a quantum system to a desired objective. This is achieved by applying coherent control fields that orchestrate the interference of quantum amplitudes, i.e., quantum coherence (*1*, *2*). Coherent control has been successfully applied for a variety of tasks (*3*), with recent emphasis on quantum technology (*4*). However, the key ingredient, coherence, remains extremely sensitive to any external perturbation. Realistically, all quantum systems are, to some extent, open and thus subject to environmental effects. Interaction between the device and the external environment generates system-environment correlations. These, in turn, effectively degrade the required agent of control coherence, leading to a detrimental effect on coherent control (*5*–*8*). Nevertheless, the inevitable “harmful” dissipation also allows redefining the possible control objective functionals by enabling nonunitary, entropy-changing transformations (*9*, *10*).

Quantum control has three tiers of theory: controllability, the existence of a solution; constructive control mechanism; and optimal control theory (OCT). Previous studies of the control of open quantum systems concentrated on constructive mechanisms of state-to-state tasks limited to specific scenarios, such as fast equilibration (*9*–*12*). In this study, we concentrate on OCT, which addresses general tasks, including quantum gates. One should differentiate state-to-state control tasks from control of gates. Such control is equivalent to simultaneous control of *N* state-to-state tasks, where *N* is the gate’s dimension. Therefore, the control field has to generate a solution for each state-to-state transformation without conflicting with the other ones. The result is a complexity that is *N*-factorial more difficult (*13*).

The second law of thermodynamics restricts the possible admissible dynamics in the control process. Work is irreversibly transformed to heat, meaning that energy change in the controller is dissipated in the environment. On the other hand, heat can flow bidirectionally from the environment to the controlled system, provided that the total entropy production is positive.

The current study explores entropy-changing control targets. The basic proposition is based on the realization that the external drive influences not only the primary system (directly) but also the dissipation induced by the environment (indirectly). We will demonstrate how the interplay between direct and indirect controls can lead to the formation of important building blocks for quantum coherent control, such as unitary gates under dissipative conditions and irreversible reset operations. In addition, we analyze the thermodynamic consequences of the open-system control processes. Rapid control protocols require additional heat dissipation to the environment, and the unitary gates are accompanied by active cooling for maintenance of high-purity systems at the cost of large entropy production.

The control relies on the intimate relationship between the isolated system (free) dynamics and the dissipative part of the dynamics. This relationship is a consequence of a global Hamiltonian $\hat{H}$, which includes a quantum description of the system, controller, and environment. Our control objective functional is defined solely in terms of system observables, which are subject to environmental influence. The environment is thermal and stationary and, therefore, uncontrollable.

Such a control process is described within the framework of open quantum systems (*14*), where the reduced description is given by an appropriate nonunitary dynamical equation of motion. Assuming negligible initial correlations between the system and the environment, the reduced dynamics are governed by a completely positive trace-preserving map (CPTP): ${\hat{\rho }}_{S}(t)=\Lambda_{t}{\hat{\rho }}_{S}(0)$ (*15*). This map is generated by the dynamical equation

$$\frac{d}{\mathrm{dt}}{\hat{\rho }}_{S}(t)=L_{t}[{\hat{\rho }}_{S}(t)]$$ (1)

The precise form of the generator is obtained by a first-principle “microscopic” derivation (see Methods). The equation is valid under a number of conditions. Primarily, weak coupling between the system and the environment is assumed. Such a restriction is justified by the appreciable efforts to isolate the experimental setups from the environment to maintain a high degree of coherence. In addition, we assume a time scale separation between the slow system and the fast environment.

The validity range of the present analysis can be defined in terms of the four typical time scales: τ_R_ the relaxation time scale, the typical system time scale τ_S_, a time scale characterizing the decay of the environment’s correlation functions (memory) τ_E_, and a time scale associated with the driving protocol τ_d_. The weak coupling regime implies that τ_R_ is much larger than τ_S_, while the Markovian character of the environment is associated with a very short τ_E_. Overall, the considered physical regime can be summarized by τ_E_ ≪ (τ_S_, τ_d_) ≪ τ_R_. We emphasize that the driving may be highly nonadiabatic, leading to τ_d_ ≈ τ_S_. For a detailed derivation and extended discussion, see Methods.

The control dynamical equation is of the Gorini-Kossakowski-Lindblad-Sudarshan (GKLS) form (*16*, *17*)

$$\frac{d}{\mathrm{dt}}{\hat{\rho }}_{S}(t)=-\frac{i}{\hbar }[{\hat{H}}_{S}(t),{\hat{\rho }}_{S}(t)]+L_{d}[{\hat{\rho }}_{S}(t)]$$ (2)

where the dissipative part ℒ*_d_* has the structure

$$L_{d}[{\hat{\rho }}_{S}]=\sum_{j}\gamma_{j}(t)({\hat{F}}_{j}(t){\hat{\rho }}_{S}(t){\hat{F}}_{j}^{†}(t)-\frac{1}{2}\{{\hat{F}}_{j}^{†}(t){\hat{F}}_{j}(t),{\hat{\rho }}_{S}(t)\})$$ (3)

Here, the Lindblad jump operator ${\hat{F}}_{j}$ constitutes eigenoperators of the free dynamical map U*_S_*(*t*) (Eq. 8), and the kinetic coefficient γ*_j_*(*t*) is real and positive. The free dynamical map is generated by the control Hamiltonian, decomposed of the bare-system drift Hamiltonian and a time-dependent control term

$${\hat{H}}_{S}(t)={\hat{H}}_{S}^{0}+\hat{V}(t).$$ (4)

The controller $\hat{V}(t)$ influences the system state both directly, through the unitary term, and indirectly, through the jump operators and kinetic coefficients of the dissipative part. As a result, the controller modifies the fixed point of the dynamical equation, formally defined by the relation $L_{t}[{\hat{\rho }}_{S}^{i.a}(t)]=0$. The dynamics aspire to lead the system to the state ${\hat{\rho }}_{S}^{i.a}(t)$. Because such a state varies in time, it is termed the instantaneous attractor. In the absence of any control ($\hat{V}(t)=0$), the system will settle to thermal equilibrium, determined by the drift Hamiltonian ${\hat{H}}_{S}^{0}$ and the bath temperature. The derivation leading to Eq. 2 guarantees a positive entropy production in the composite system, including the system, controller, and environment. The indirect influence of the driving on the dissipation paves the way to the control of the open-system dynamics.

The dependency of the dissipation on the control suggests the following iterative control procedure:

1)Guess a control field, *V*(*t*) with *t* ∈ [0, *t_f_*], and apply it to calculate an explicit solution of the system’s free dynamics U*_S_*(*t*) (Eq. 5).

2) Construct the master equation according to Eq. 2.

3) Calculate the system’s dynamics ${\hat{\rho }}_{S}(t).$

4) Using the final state, ${\hat{\rho }}_{S}(t_{f})$, evaluate the control objective functional, defined according to the specific control task at hand (see below).

5) Use the evaluated control objective functional to update the control field.

In step 2, we construct U*_S_*(*t*) from the unitary evolution operator: $U_{S}(t,0)[•]={\hat{U}}_{S}(t,0)•{\hat{U}}_{S}^{†}(t,0)$, generated by the time-dependent Hamiltonian ${\hat{H}}_{S}(t)$

$$i\hbar \frac{\partial }{\partial t}{\hat{U}}_{S}(t)={\hat{H}}_{S}(t){\hat{U}}_{S}(t)$$ (5)

with ${\hat{U}}_{S}(0)=\hat{I}$. For specific control protocols, Eq. 5 yields closed-form solutions, which can be extended for slow deviations from these protocols, using the inertial theorem (*18*). However, a general analytical solution requires overcoming a time-ordering procedure (*19*).

In this study, we bypass the time-ordering obstacle using a numerical solution for the free dynamics (Eq. 5). This procedure generates the eigenstates of the time-evolution operator

$${\hat{U}}_{S}(t)|\phi_{n}(t)\rangle =e^{-i\epsilon_{n}(t)}|\phi_{n}(t)\rangle$$ (6)

From ∣ϕ*_n_*(*t*)〉, we construct the eigenoperators

$${\hat{F}}_{j}(t)=|\phi_{n}(t)\rangle \langle \phi_{m}(t)|$$ (7)

where *j* = *N*(*n* − 1) + *m*. These satisfy the eigenvalue-type relation with respect to the free propagator

$$U_{S}(t,0){\hat{F}}_{j}(t)={\hat{U}}_{S}(t,0){\hat{F}}_{j}(t){\hat{U}}_{S}^{†}(t,0)=e^{-i\theta_{j}(t)}{\hat{F}}_{j}(t)$$ (8)

where θ*_j_*(*t*) = ε*_n_*(*t*) − ε*_m_*(*t*) are the corresponding phases. They determine the effective instantaneous Bohr frequencies of the driven system ω*_j_*(*t*) = *d*θ*_j_*(*t*)/*dt*. The noninvariant eigenoperators occur in conjugated pairs with complex conjugate eigenvalues and constitute the transition operators between the instantaneous eigenstates of ${\hat{U}}_{S}(t)$. Concurrently, eignoperators with θ*_j_*(*t*) = 0 are the instantaneous projection operators, {∣ϕ*_m_*(*t*)〉〈ϕ*_m_*(*t*)∣}.

The remaining task for obtaining the control dynamical Eq. 2 (step 2) is to calculate the kinetic coefficients {γ*_j_*(*t*)}. The fact that the jump operators associated with a certain transition are related by a detailed balance relation motivates relabeling the kinetic coefficients *k*_*i*,↑_(*t*), *k*_*i*,↓_(*t*), where *i* = *j*/2 corresponds to a conjugate pair of eigenoperators. In the weak coupling limit and under Markovian dynamics, these coefficients can be calculated from the Fourier transform of the environmental correlation functions with an instantaneous frequency ω*_j_*(*t*) (*20*, *21*). The dynamical equation solution yields ${\hat{\rho }}_{S}(t)$ (step 3), which allows for calculating the control objective functional. Last, the objective is used to update the control field (steps 4 and 5). Steps 2 to 5 are reiterated until convergence.

## RESULTS

### Model

To demonstrate the algorithm, we choose a model for which the free dynamics are completely controllable and can scale from a two-level system (TLS) to an *N*-level system. The single-mode Bose-Hubbard (BH) model (*22*) serves this task. The model was originally intended to describe *N* particles in a double-well potential. It is isomorphic to a Hamiltonian composed of angular momentum operators with *j* = *N* + 1, where *j* is the total angular momentum

$${\hat{H}}_{S}^{0}=u{\hat{J}}_{z}^{2}+\Delta {\hat{J}}_{x}$$ (9)

Here, ${\hat{J}}_{x}$ represents the hopping operator, and ${\hat{J}}_{z}^{2}$ is the on-site interaction operator. We set *u* = 2Δ/*j*, for which the dynamics are classically chaotic (*23*). The control Hamiltonian is chosen as

$$\hat{V}(t)=\epsilon (t){\hat{J}}_{z}$$ (10)

where ${\hat{J}}_{z}$ is the control operator and ϵ(*t*) is the control field.

The driven Bose-Hubbard system has complete controllable free dynamics. Such controllability arises from the fact that the commutators of the drift Hamiltonian (Eq. 9) and the control (Eq. 10) generate the full algebra (*24*, *25*). Moreover, the same control Hamiltonian is scalable to an arbitrary *N*-level system characterized by a *SU*(*N*) Lie algebra. For spin half, it reduces to the qubit Hamiltonian, where the drift Hamiltonian is in the ${\hat{\sigma }}_{x}$ direction.

The dynamics of the closed-system evolution operator, ${\hat{U}}_{S}(t)$ (Eq. 5), are integrated numerically by a Chebychev propagator (*26*). At each intermediate time, ${\hat{U}}_{S}(t)$ is diagonalized to obtain the time-dependent orthonormal set of jump operators, $\left\{{\hat{F}}_{j}(t)\right\}$ (Eq. 7), and Bohr time-dependent frequencies {ω*_j_*(*t*)}. The jump operators are then used to compute the Liouvillian dynamics in the interaction picture. Using the Liouvillian superoperator, the full dissipative equation of motion (Eq. 2) was propagated numerically to obtain ${\hat{\rho }}_{S}(t)$, using a Newtonian polynomial method (*27*).

The environment was chosen as a bosonic Ohmic bath composed of an ensemble of harmonic modes with a spectral density *J*(ω) = *c*ω^2^ (see details in section Methods), where *c* is a scaling constant that maintains the units. Such a choice corresponds to an interaction with the electromagnetic field or a phonon bath. For each term in the sum of Eq. 3, the corresponding kinetic coefficients are functions of the Bohr frequency ω*_j_*(*t*)

$$k_{j,↑}(t)=g^{2}\omega_{j}(t)J(\omega_{j}(t))N(\omega_{j}(t))=k_{j,↓}(t)e^{-\hbar \omega_{j}(t)/k_{B}T}$$ (11)

where *N*(ω) = 1/(*e*ℏω/*k*B*T* − 1) and *g* is the system-environment coupling strength. For the numerical analysis, we set the parameters such that *g*^2^*c* = 10^4^ in atomic units, and system-bath coupling operator is proportional to ${\hat{H}}_{I}={\hat{J}}_{y}⊗\hat{B}$, where $\hat{B}$ is the bath interaction operator (for the exact form, see Eq. 26).

The control scheme used to evaluate the optimal field is a simplified version of the Chopped RAndom Basis set optimization (CRAB) algorithm (*28*, *29*). For each task, a cost function was defined (see below). The control field (Eq. 10) is given by

$$\epsilon (t)=\text{exp}\;(-{\left(\frac{t-\tau /2}{2\sigma }\right)}^{2})\sum_{k=1}^{M}c_{k}\text{sin}(\nu_{k}t)$$ (12)

where σ is the pulse width, τ is the target control time, and ν*_k_* is a set of *M* frequencies. The coefficient *c_k_* was varied to optimize the cost function, using a standard quasi-Newton algorithm. In the present study, the amplitude of the control field was not constrained. Nevertheless, one can include additional constraints within this CRAB-like method, such as a total pulse energy restriction of the form λ ∫ ϵ(*t*)^2^*dt* or entropy generation. The CRAB family of methods achieves the control objective, relying only on the performance at final time *t*_f_. More comprehensive methods, such as optimal and local control, and other gradient methods could be used to enhance the efficiency and precision of the search. However, these will demand a more careful treatment, both analytically and numerically. The advantage of preselecting a fixed pallet of control frequencies in Eq. 12 is that they can be chosen to fit experimental constraints (*29*).

### Control

To illustrate the control scheme, we first demonstrate state-to-state entropy-changing tasks and proceed by analyzing dynamical map’s control. In all studied cases, the control landscape was found to contain traps, meaning that suboptimal minima exist. We overcame this difficulty using hundreds of realizations with different random initial guesses for the field. The presented solutions are the best for this set.

#### Heating and cooling

The hallmark of open-system control is a change in the system’s von Neumann entropy

$$S=-k_{B}\mathrm{tr}({\hat{\rho }}_{S}\text{log}\;({\hat{\rho }}_{S}))$$ (13)

(atomic units are used throughout the study). Because unitary control necessarily preserves the eigenvalues of ${\hat{\rho }}_{S}$, S must be constant for isolated systems. Thus, the change of S is a clear indication of interaction with an external environment. This property motivates the choice of the entropy S as our cost functional for the state-to-state control objective. Heating or cooling is defined by an increase or decrease of the system’s von Neumann entropy, respectively. For the demonstration, we choose an initial thermal state ${\hat{\rho }}_{S}^{i}$ with inverse temperature β ≡ 1/*k*_B_*T* = 1/ℏΔ.

In open-system dynamics, the system and the environment entropies vary. The total amount of entropy produced can be evaluated by integrating the entropy production rate

$$\Sigma^{U}(t)\equiv -\frac{d}{\mathrm{dt}}D({\hat{\rho }}_{S}|{\hat{\rho }}_{S}^{i.a})=-k_{B}\mathrm{tr}(L_{t}[{\hat{\rho }}_{S}]\text{log}\;{\hat{\rho }}_{S})+k_{B}\mathrm{tr}(L_{t}[{\hat{\rho }}_{S}]\text{log}\;{\hat{\rho }}_{S}^{i.a})$$ (14)

where D is the divergence and ${\hat{\rho }}_{S}^{i.a}$ is the time-dependent instantaneous attractor (*20*), which satisfies $L[{\hat{\rho }}_{S}^{i.a}]=0$. By integrating Eq. 14 over the protocol duration, one obtains the total entropy production.

##### Heating

Our current control task is to heat the system as much as possible. For an *N*-level system, this task defines the target state as the microcanonical distribution ${\hat{\rho }}_{S}^{f}=\hat{I}/N$, with the maximal entropy S^max^ = log *N*.

**Fig. 1.**
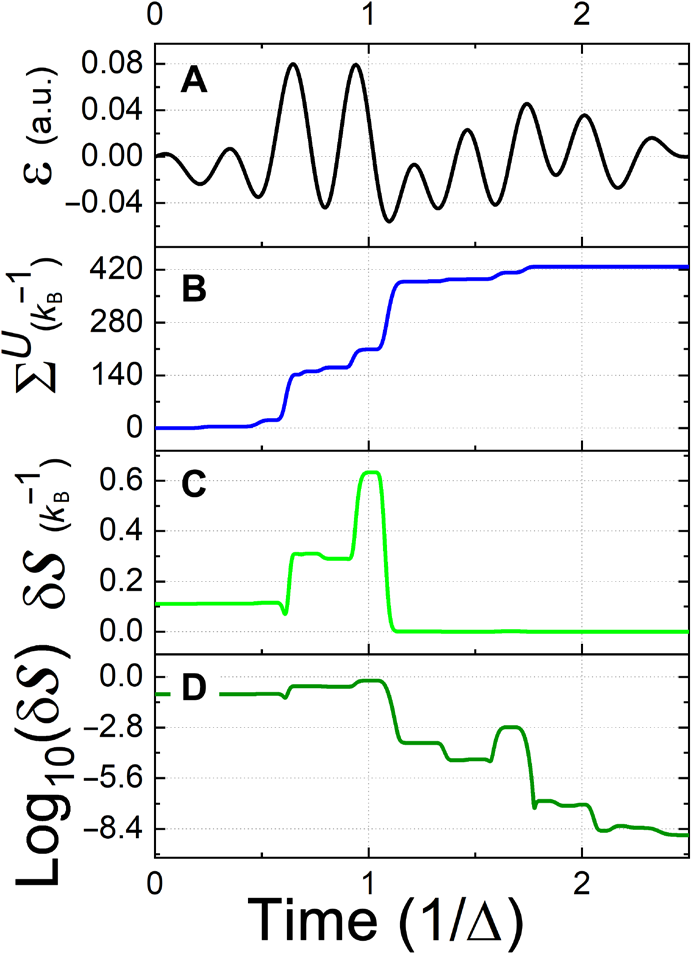
Controlled heating of the TLS. Time is defined in units of inverse frequency Δ (Eq. 9): (**A**) The optimized control field. (**B**) Accumulated entropy production, obtained by the time integration of the entropy production rate in Eq. 14. (**C**) Divergence of the system’s entropy from its maximal value in a linear scale. (**D**) The same as (C) on a logarithmic scale. a.u., atomic units.

Figure 1 demonstrates a controlled heating task for a TLS, which corresponds to the BH model (Eq. 9), with *j* = 1/2. We find that, initially, coherence is generated, and the system’s entropy decreases. While at the final stage, the dissipation of coherence is accompanied by substantial heating, leading to an entropy production of about three orders of magnitude larger than the system’s change in entropy. This result complies with the fact that the optimization was performed only with respect to the system’s entropy, while the dissipative entropy generation was not constrained.

The nonmonotonic behavior of the system entropy arises from the change in its energy levels due to the drive. Heuristically, when the energy gap between the two levels is large with respect to *k*_B_*T*, the energy flows from the system to the bath and vice versa. Hence, the driving may indirectly modify the direction of the system entropy flow and cause such nonmonotonic behavior. As required by the thermodynamic laws, the total entropy production remains positive even when the system’s entropy decreases.

The protocol was calculated by performing an optimization over the control space, which corresponded to *M* = 20 field frequencies (see Eq. 12). In addition, the time scale of the control pulse was chosen to be inversely related to the TLS energy difference 2π/Δ, which was much shorter than the chosen natural, spontaneous decay rate, given by 10^−4^/Δ. This boost in performance stems from the dependence of the kinetic coefficient, {γ*_j_*} (Eq. 3), on the driving parameters. The indirect control over the kinetic coefficients leads to the maximal entropy state with a precision of 10^−9^.

**Fig. 2.**
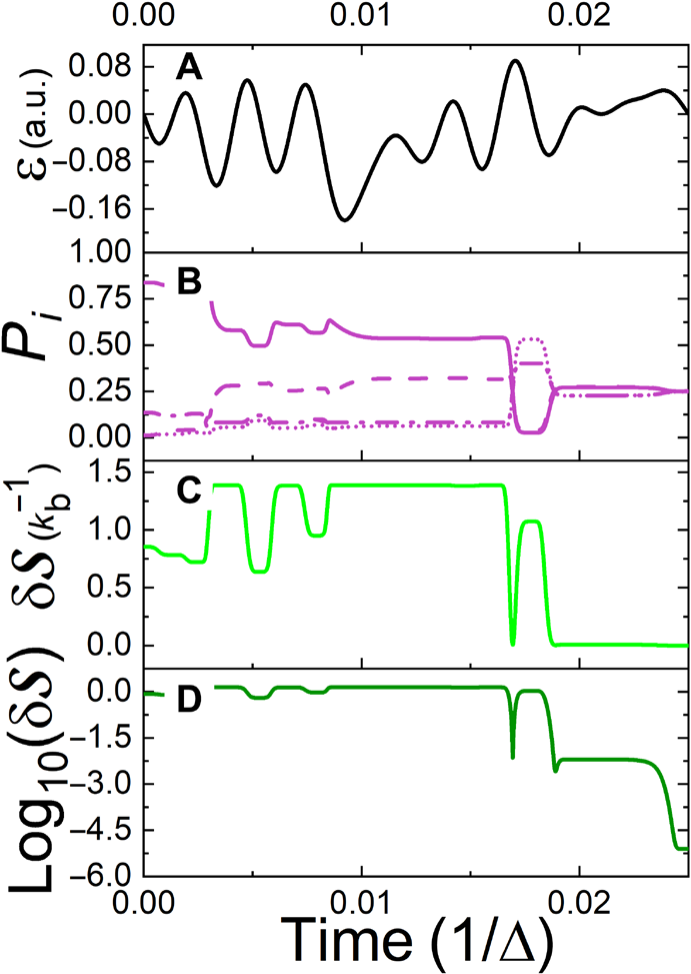
Controlled heating of the four-level system (*N* = 4). (**A** to **D**) As in Fig. 1, apart from (B), which presents the population of the system’s four states as a function of time. At the final time, a uniform distribution is obtained, corresponding to an infinite-temperature thermal state.

The same maximum entropy objective has been used for four levels, corresponding to the BH with *j* = 3/2 (see Fig. 2). The target of maximum entropy is reached with in a relative error of 10^−5^ compared to its maximal value of S^max^ = ln (*N*). This is a notable reduction in the accuracy with respect to the TLS case. The control duration for the four-level system is characterized by a short protocol duration relative to the TLS (see Fig. 2). The calculation, complexity of the control, increases with the number of possible interference paths and therefore grows exponentially with the number of levels and control duration. This fact ultimately influences the optimization procedure, as finding the optimal control protocol becomes more demanding with the increase in Hilbert space size. As a consequence, the precision of the control objective δS is reduced along with the effective time window for which a solution can be found.

##### Cooling

**Fig. 3.**
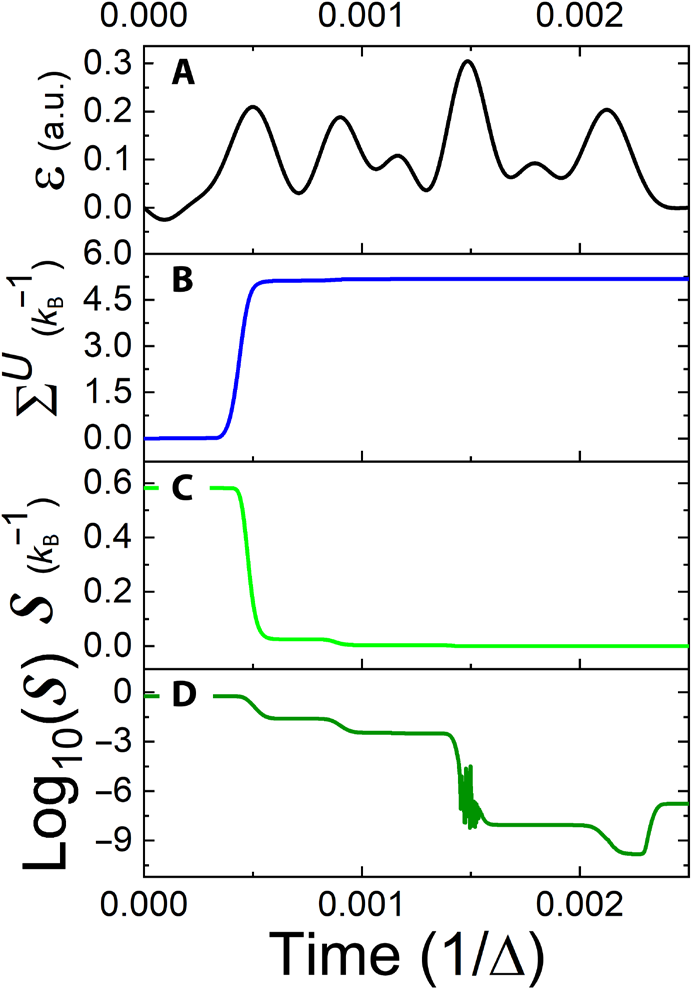
Controlled cooling of the TLS: Designation similar to Fig. 1. (**A** to **D**) The target of control, the system’s entropy, is displayed in (C) and (D) on linear and logarithmic scales, respectively.

From a formal algorithmic point of view, cooling is almost identical to heating but with a modified objective. Here, the goal is to minimize the system’s entropy, and therefore, the final target state is pure, satisfying ${\left({\hat{\rho }}_{S}^{f}\right)}^{2}={\hat{\rho }}_{S}^{f}$. Moreover, in both processes, once the target state is reached and coherence vanishes, the expectation value of the controller vanishes, resulting in a strictly uncontrollable system. Physically, however, the two processes differ substantially from one another. The asymmetry originates from the third law of thermodynamics, which implies that the resources required for cooling to zero entropy diverge (*30*–*32*). Figure 3 presents the controlled cooling of a TLS. The objective, which is the minimization of the final-state entropy, is obtained with high accuracy. However, we find that achieving extremely low entropy values typically requires large control field amplitudes, leading to numerical instabilities. A possible remedy is to introduce an extra term in the cost function, preventing the abusive use of resources by the control.

**Fig. 4.**
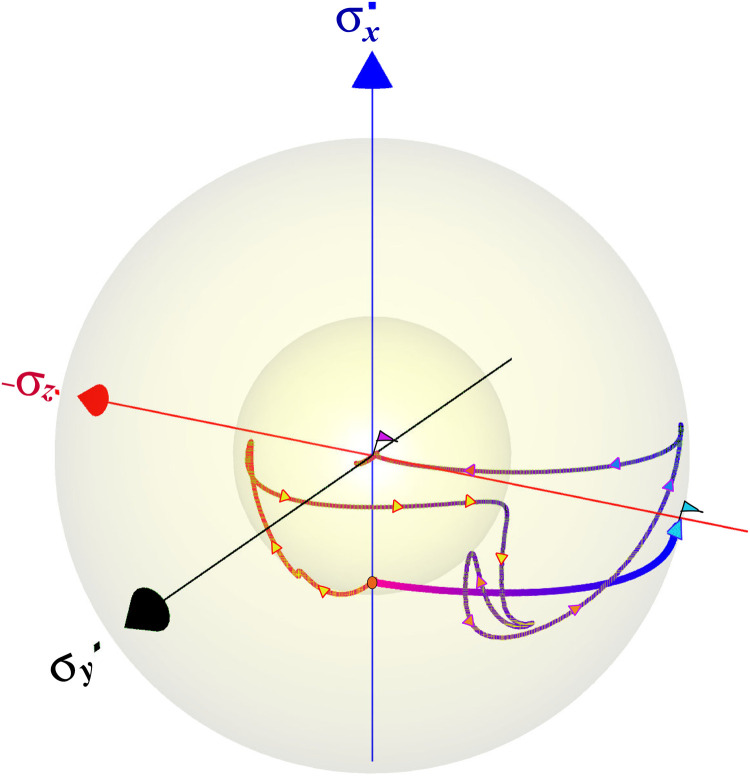
The control trajectories of the heating and cooling solutions displayed on the Bloch sphere. The common initial thermal state is designated by an orange dot on the *x* axis. The cooling trajectory monotonically approaches a pure state on the surface of the Bloch sphere in the σ*_z_* direction. The heating trajectory transverses a more complex trajectory, first exhibiting an increase in purity (the inner sphere represents the initial purity) and at the final stage, approaching a completely mixed state at the origin. These trajectories constitute one of many possible solutions to the optimal control problem.

A control trajectory in the Bloch sphere for optimal heating and cooling processes is shown in Fig. 4. The projection of the instantaneous ${\hat{\rho }}_{S}(t)$ on the three Pauli operators is shown in the Bloch sphere. In this geometrical representation of the TLS, the state’s distance from the origin represents its purity. The cooling and heating trajectories initialize at the same thermal state, designated by an orange dot. Heating brings the initial state to the origin via a trajectory that passes through the high-radius region, corresponding to intermediate states with high purity. Cooling is achieved by a more direct path to an almost final pure state. Comparing the control protocols, the cooling protocol requires higher instantaneous power compared to the heating process. This results in an increase in the effective Rabi frequency for cooling, which agrees with the overshoot observed in the shortcut to equilibration protocols (*9*, *10*).

#### Control of dynamical maps

##### Generation of a CPTP dynamical map

${\hat{\rho }}_{S}^{f}=\Lambda {\hat{\rho }}_{S}^{i}$ constitutes a more stringent control task because a map must transform any arbitrary initial state to a corresponding target state (*15*). The dynamical map can be diagonalized into *N*^2^ independent invariant eigenoperators, where *N* is the dimension of the Hilbert spaces. The eigenvalues assume the following form: *e*^*i*Θ*_j_*^, where, for unitary maps, Θ*_j_* is real (see Eq. 8) and where Θ*_j_* = θ*_j_*(*t_f_*), for nonunitary maps, θ*_j_* is complex. As a result, the map transformation can be fully characterized using a complete operator basis along with the scalar product: $\left(\hat{A},\hat{B})=\mathrm{tr}(\hat{A}{\hat{B}}^{†}\right)$. For example, in the qubit case, we can express the state using the set of Pauli operators and identity $\left\{\hat{I},{\hat{\sigma }}_{x},{\hat{\sigma }}_{y},{\hat{\sigma }}_{z}\right\}$. The map Λ can therefore be expressed in terms of a 4 by 4 matrix (*N*^2^ = 4).

Two extreme cases are studied: a reset map Λ_R_ and a unitary map Λ_U_. The reset map transforms any initial state to a single target state. Specifically, considering an arbitrary initial state

$${\hat{\rho }}_{S}^{i}=\frac{1}{2}\hat{I}+\sum_{j=x,y,z}c_{j}{\hat{\sigma }}_{j}$$ (15)

The chosen map transforms any state to a pure state in the *x* direction

$${\hat{\rho }}_{S}^{f}=\frac{1}{2}(\begin{array}{cc} 1 & -1\\ -1 & 1 \end{array})=\frac{1}{2}(\hat{I}-{\hat{\sigma }}_{x})$$ (16)

In the operator space, spanned by $\left\{\hat{I},{\hat{\sigma }}_{x},{\hat{\sigma }}_{y},{\hat{\sigma }}_{z}\right\}$, the associated transformation is represented by the nonunitary matrix

$$\Lambda_{R}=(\begin{array}{cccc} 1 & 0 & 0 & 0\\ 0 & -1 & -1 & -1\\ 0 & 0 & 0 & 0\\ 0 & 0 & 0 & 0 \end{array}\;)$$ (17)

**Fig. 5.**
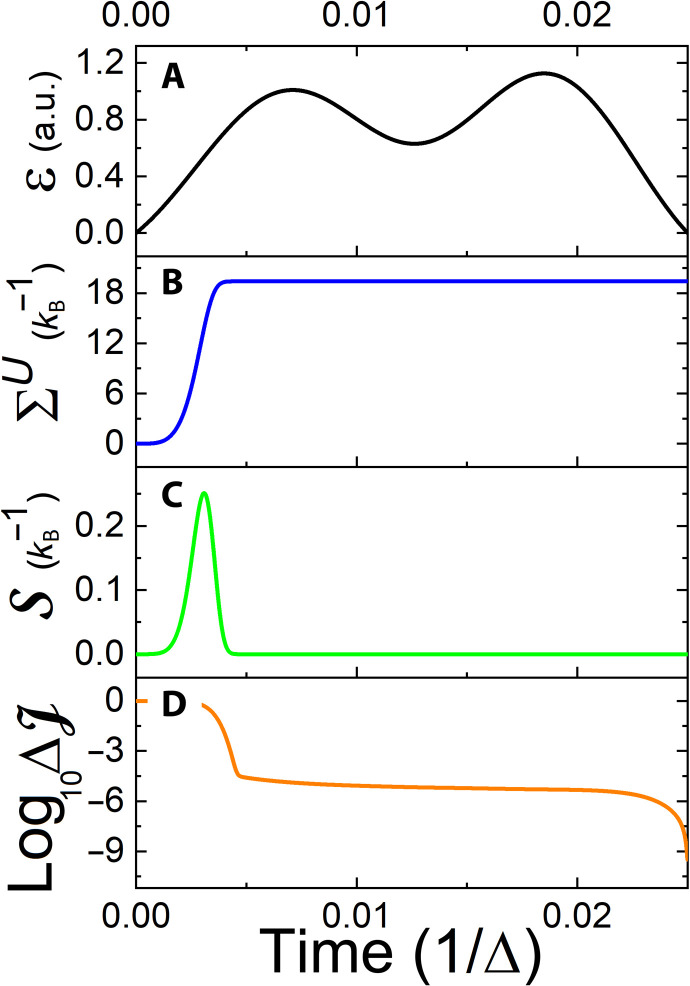
Controlled reset transformation: Transformation of an arbitrary qubit state to a final pure state ${\hat{\rho }}_{S}^{f}$ (Eq. 16). (**A**) Time-dependent control field. (**B**) Entropy production. (**C**) System’s entropy. (**D**) Deviation of the control objective in a logarithmic scale as a function of time.

To determine the generating field ϵ(*t*) (Eq. 10), a complete set of initial states $\left\{{\hat{\rho }}_{S}^{i,k}\right\}$ is used and optimized to reach the same target state ${\hat{\rho }}_{S}^{f}$. The accuracy of the transformation is then evaluated by the control objective functional, the trace distance

$$J=\sum_{k}^{N^{2}-1}\mathrm{tr}\{{\hat{\rho }}_{S}^{f,k}{\hat{\rho }}_{S}^{f}\}$$ (18)

where ${\hat{\rho }}_{S}^{f,k}=\Lambda {\hat{\rho }}_{S}^{i,k}$. We can exclude the identity in Eq. 18 because it is preserved in the CPTP map. Figure 5 demonstrates the reset transformation. As shown in Fig. 5C, at initial and final times, the systems’ states are pure, while at intermediate times, an increase in entropy indicates the necessary temporary transition to a mixed state. The obtained mechanism of the reset process can be divided into two stages: At the beginning, we witness an entropy increase, indicating a memory loss of the initial state. This is followed by a purification of the mixed-state rotation to the desired direction at the final stage.

Figure 5D presents the deviation of the system’s objective functional from its maximal value in a logarithmic scale. An initial rapid reduction in the deviation brings the system to a precision of ΔJ = 10^−5^. This is followed by a final stage, providing an additional accurate kick, which drives the system to the target state and to deviations of up to 10^−9^. Crucially, we also explicitly verified that the obtained control field transforms any randomly picked pure and nonpure state into the target state, which is indeed the manifestation of the reset transformation. Note that the meaning of such a reset transformation constitutes an ultimate cooling process of the system. That is, the obtained field cools the system effectively from any initial state to *T* ≈ 0. As expected, because no restriction was imposed on the entropy production rate, the thermodynamic cost of the reset process, exhibited in Fig. 5B, is well above its theoretical bound given by the Landauer limit (*33*).

#### Unitary maps

We next tackle the task of inducing a unitary transformation under dissipation (*34*–*36*). In this case, *N*^2^ independent eigenoperators of the unitary map have to be transformed along with their correct phases. This requires a simultaneous *N*^2^-level state-to-state transformation, which is factorially more difficult to achieve. Otherwise, a classical computer could compile polynomially any quantum gate (*13*).

The chosen demonstrative transformations are the one-qubit Hadamard Λ_U_ and two-qubit entangling gate $\Lambda_{\sqrt{S}}$. Because an entangling gate and the full set of single-qubit rotations form a universal set of quantum gates, combining these control protocols can form an arbitrary unitary gate (*37*–*40*). These transformations can be incorporated in noisy quantum information processing, producing effective unitary single-qubit and two-qubit gates under dissipation.

A single-qubit gate corresponds to a rotation in the Bloch sphere and can be expressed as a superoperator in $\left\{\hat{I},{\hat{\sigma }}_{x},{\hat{\sigma }}_{y},{\hat{\sigma }}_{z}\right\}$ the operator basis. Specifically, the Hadamard gate is given by

$$\Lambda_{U}=(\begin{array}{cccc} 1 & 0 & 0 & 0\\ 0 & 0 & 0 & -1\\ 0 & 0 & -1 & 0\\ 0 & -1 & 0 & 0 \end{array})$$ (19)

**Fig. 6.**
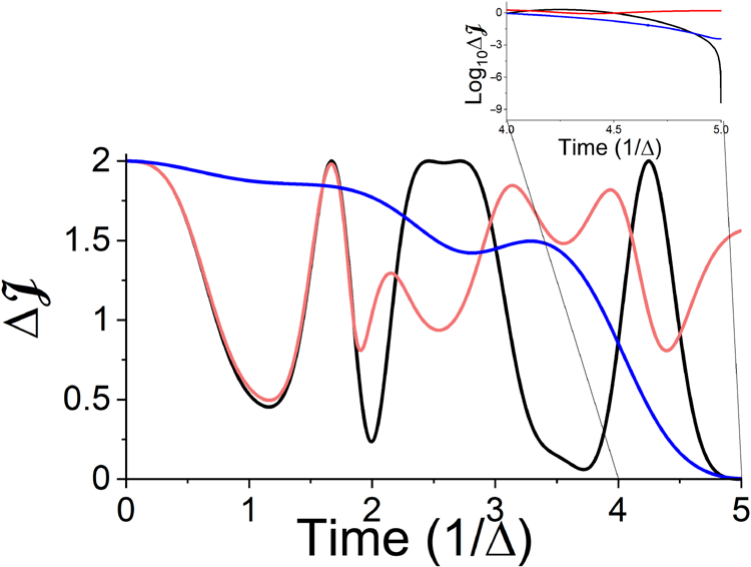
Controlled Hadamard gate: Deviation of the objective functional (Eq. 18) as a function of time for the controlled Hadamard gate. Linear and (inset) logarithm scales. Optimal transformation under dissipation-free propagation (black). The dynamics of the transformation with the same field, subject to the environment (relaxation time of τ = 10^−11^s) (red). Optimal dynamics for the open system (blue).

**Fig. 7.**
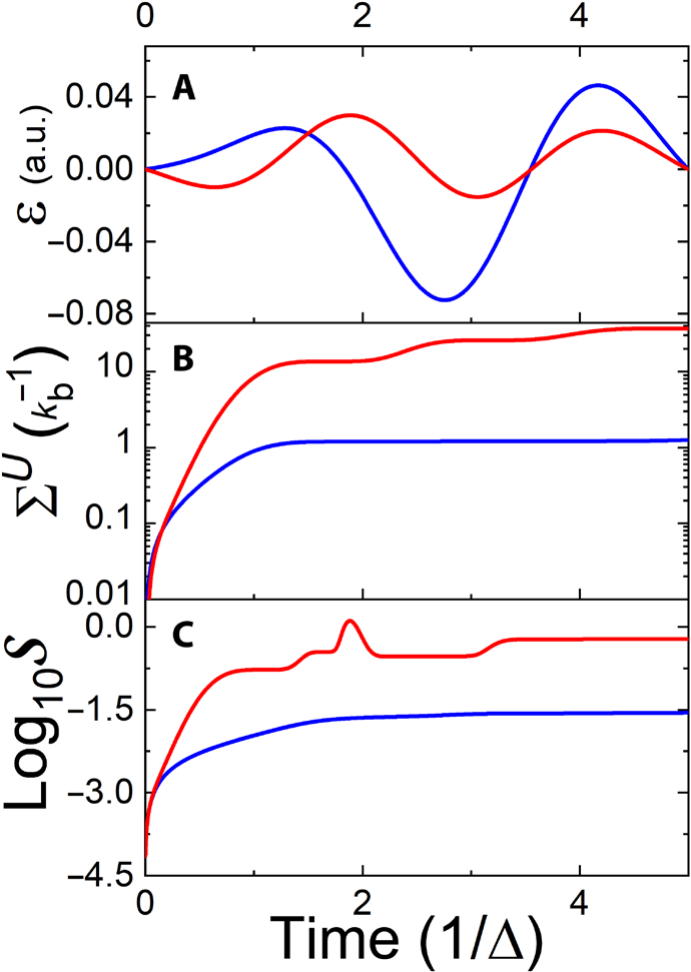
Controlled Hadamard gate. (**A**) Control field, (**B**) entropy production, and (**C**) system’s entropy as a function of time for the Hadamard transformation (Eq. 19). Color designation is the same as in Fig. 6. The field for the open-system dynamics is enlarged by a factor of 10 to enable comparison.

The algorithm leading to the optimal field is similar to the one described in the “Control of dynamical maps” section. Namely, we initialized the system with a complete set of pure density operators $\left\{{\hat{\rho }}_{S}^{i,k}\right\}$ and chose the cost function as the sum of trace overlaps between the final and target states. The results are displayed in Figs. 6 and 7. To obtain a measure of the validity and robustness of the protocol, we explored three different scenarios:

1) First, the optimization was performed for an isolated system. Such a protocol coincides with the conventional closed-system unitary control. The results of this optimization are presented in the black curves in the figures. One can see that the transformation is achieved with the expected high accuracy.

2) The same optimal field in scenario 1 was applied to the open quantum system. The results are presented in red. It can be observed that, while at early stages, the dynamics seem similar, they later deviate notably. The final precision, ΔJ, is well above the reliable operational threshold of feasible gates. The degradation in precision is accompanied by an undesired increase in entropy (Fig. 7), stemming from the coupling with the environment.

3) Last, the optimization was generated from scratch, taking into account the full open-system dynamics. The associated results are presented by blue curves. Accounting for the external dissipation allows the control to cope with the environmental noise. Despite the strong decoherence, the unitary transformation precision is below the threshold of ΔJ = 10^−3^, well within the acceptable specs of feasible quantum gates. The presence of a relatively strong system-environment coupling leads to the generation of entropy. Nevertheless, it is reduced with respect to the reference protocol, and the entropy leak is later suppressed by the field.

Unexpectedly, we observe that the required control field amplitude for the open-system dynamics is appreciably lower than the free dynamics control field (see Fig. 7). As a consequence, the total energy used by the optimal field is smaller by two orders of magnitude.

**Fig. 8.**
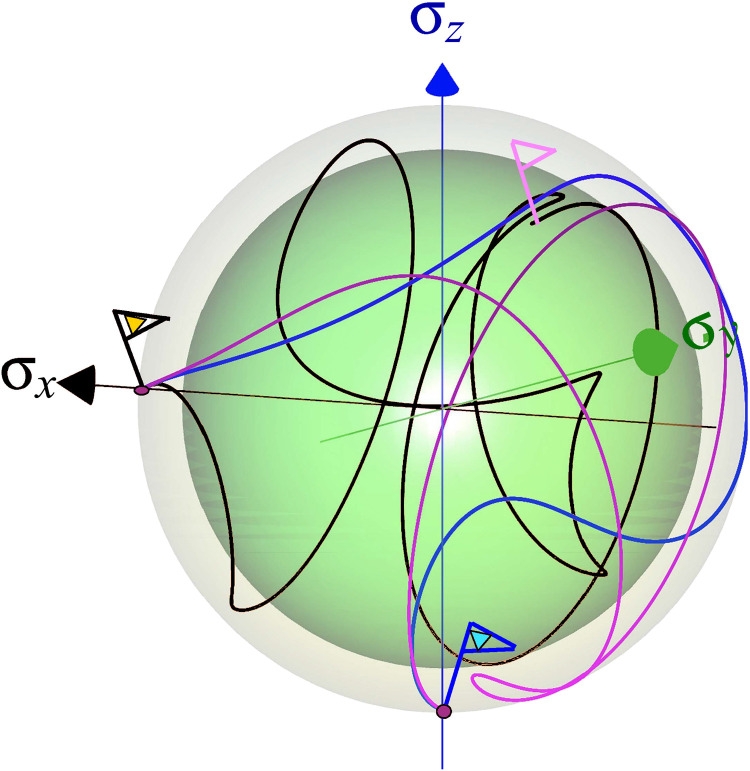
Control trajectory for the Hadamard transformation: Displaying the transition from the *x* direction to the −*z* direction (indicated by flags). The qubit state under unitary control with no environmental coupling is represented by the blue curve, while the purple line depicts the optimal state trajectory, obtained from the control protocol that includes the environmental influence (corrected protocol). The black trajectory corresponds to the state dynamics under a unitary field, not accounting for the environmental effect on the open system. Overall, we find that the optimal trajectory (purple) resides on the surface of the Bloch sphere. Trajectories entering the inner sphere represent loss of purity associated with uncorrected transformation (the final state is indicated by a pink flag). Other orthogonal directions exhibit a similar pattern.

Figure 8 compares the dynamical map-generated trajectories associated with the cases 1 to 3. The trajectory of the unitary control protocol under noise (procedure 2) misses the target, while the isolated dynamics (procedure 1) and the optimized open-system protocol (procedure 3) lead to the desired final state. The trajectory is close to the surface of the sphere and, therefore, is close to a unitary path. The possible mechanism resembles decoherence control by tracking (*41*).

Last, we find the expected result, i.e., the control precision degrades when the dissipation increases. This can be observed in fig. S2, where the control objective was studied with increased system bath coupling. The precision, nevertheless, agrees better by at least an order of magnitude with the uncorrected control protocol. The two-qubit gate optimizations show similar behavior. Note that even for the strongest coupling presented, the weak coupling condition is still maintained, and the typical time to achieve thermal equilibrium is larger by three orders of magnitude relative to the transformation time.

#### Two-qubit gates

A universal set of quantum gates can be obtained by adding an entangling gate to the single-qubit rotation gates. We demonstrate this task using the following drift Hamiltonian

$${\hat{H}}_{S}^{0}=\hbar \omega_{1}{\hat{\sigma }}_{1}^{z}+\hbar \omega_{2}{\hat{\sigma }}_{2}^{z}=\hbar (\begin{array}{cccc} -\omega_{1}-\omega_{2} & 0 & 0 & 0\\ 0 & \omega_{1}-\omega_{2} & 0 & 0\\ 0 & 0 & -\omega_{1}+\omega_{2} & 0\\ 0 & 0 & 0 & \omega_{1}+\omega_{2} \end{array})$$ (20)

and control term

$$\hat{V}(t)=\hbar \epsilon (t)({\hat{\sigma }}_{1}^{+}{\hat{\sigma }}_{2}^{-}+{{\hat{\sigma }}^{+}}_{2}{\hat{\sigma }}_{1}^{-})=\hbar \epsilon (t)(\begin{array}{cccc} 1 & 0 & 0 & 0\\ 0 & 0 & 1 & 0\\ 0 & 1 & 0 & 0\\ 0 & 0 & 0 & 1 \end{array})$$ (21)

The entangling two-qubit transformation is taken to be the square root of the swap gate $\Lambda_{\sqrt{S}}={\left({\hat{W}}_{\sqrt{S}}^{†}\right)}^{T}\hat{⊗}W_{\sqrt{S}}$, where

$${\hat{W}}_{\sqrt{S}}=(\begin{array}{cccc} 1 & 0 & 0 & 0\\ 0 & \frac{1+i}{2} & \frac{1-i}{2} & 0\\ 0 & \frac{1-i}{2} & \frac{1+i}{2} & 0\\ 0 & 0 & 0 & 1 \end{array})$$ (22)

and $\hat{W}$ operates in the two-qubit Hilbert space. In isolated conditions, this transformation addresses only the two-qubit subspaces ∣01⟩ and ∣10⟩. The transformation then becomes a rotation in the *SU*(2) subalgebra of the four-level algebra *U*(4). In the dissipative case, the control must minimize population leakage to other states. The optimization was performed by a similar method to the one described in the “Control of dynamical maps” section.

**Fig. 9.**
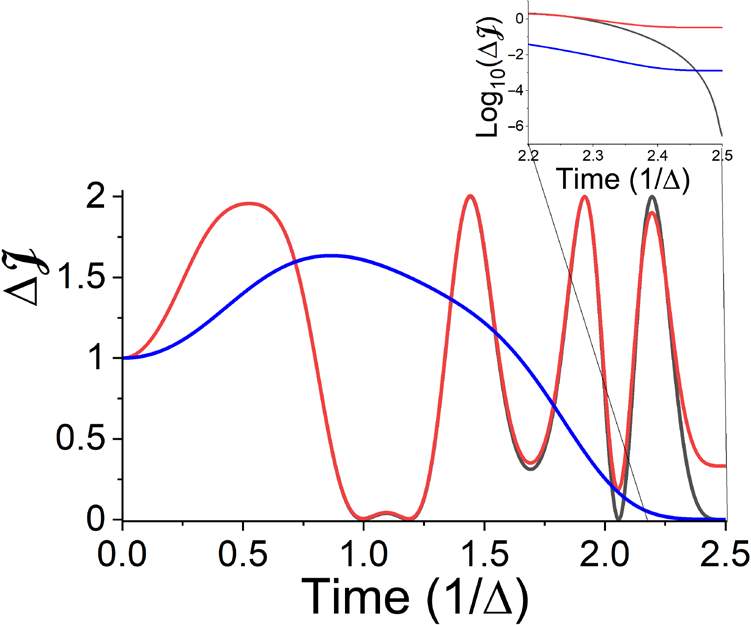
Controlled square root swap gate (Eq. 22): Transformation precision as a function of time. Designation is similar to Fig. 6. For the demonstration, we chose ω_2_ = 1.1ω_1_ with ω_1_ = Δ = 3 × 10^−3^ a.u.

The control protocol has been studied using the same three schemes used for the reset transform. Figure 9 displays the objective functional ℐ as a function of time. The figure’s inset shows the deviation of the objective from the target state on a logarithmic scale during the final protocol stage. Uncorrected for the environmental influence within the control, the system deviates considerably from the objective, while a complete optimization, including the environmental influence, reaches the objective with high fidelity.[Fig F10]

**Fig. 10. F10:**
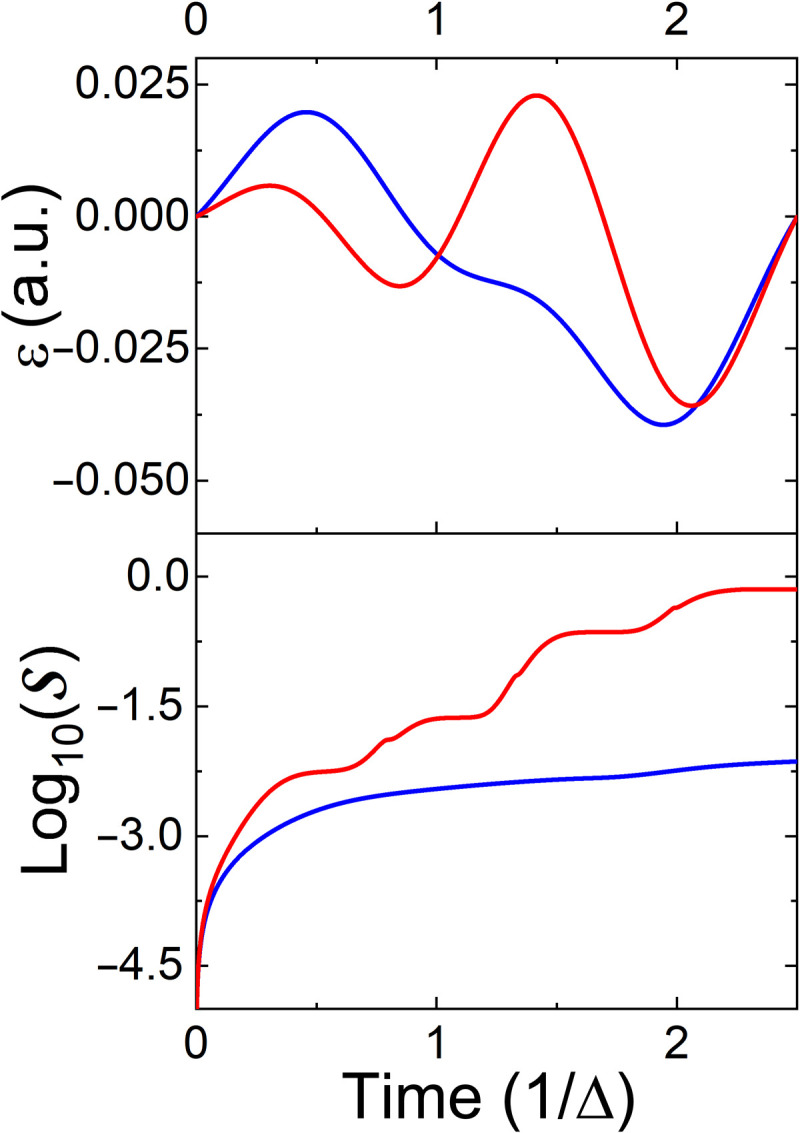
Two-qubit gate. (**Top**) Optimal field as a function of time. (**Bottom**) Entropy as a function of time. Designation is similar to Fig. 7.

The control trajectories can be graphically depicted by evaluating the operators of the *SU*(2) algebra and characterized by the generalized purity. This measure is defined as the purity of the projected state on the *SU*(2) algebra (*42*). Using such a representation, a similar picture to Fig. 8 emerges in Figure 10. The successful gates maintain constant generalized purity, while the uncontrolled ones degrade the generalized purity as a result of the coupling with the environment.

## DISCUSSION

Control targets are closely linked to the resources required to achieve them. We can consider three types of resources that are closely linked: quantum interference, thermodynamic, and algorithmic.

### Quantum interference

The resource of coherent control constitutes all possible interference pathways between initial and final states. For an *N*-level system, their number is linear in the spectral bandwidth of the control pulse. Moreover, as the intensity increases, the number of interference possibilities increases exponentially because of multicycle transitions.

The complexity of the control tasks is minimal for binary state-to-state transformations of isolated systems, requiring only a wave function description (scales as *N*). In comparison, open-system state-to-state control requires using a density operator formalism (∼*N*^2^). By diagonalizing the open-system density operator, we can infer that such a transformation requires at least *N* − 1 times more computational resources than the isolated case.

Unitary gates for isolated systems demand factorially more control resources because they require *N* independent state-to-state transformations. Within this hierarchy, the most difficult control task is to construct a quantum gate for an open quantum system (see the “Control of dynamical maps,” “Unitary maps,” and “Two-qubit gates” sections). Together, the dimension of this transformation is squared (∼*N*^2^) and requires an independent control solution for each eigenoperator of the gate. This is the primary problem addressed in this study.

### Thermodynamic

The various control tasks are accompanied by a thermodynamic cost. This cost is related to the energy change of the controller (work) or, equivalently, the integrated pulse energy. Practically, this work is dissipated to the environment directly from the controller or through the open quantum system. Such spontaneous energy flow is the manifestation of the second law of thermodynamics.

For the open-system control task studied here and, in particular, for unitary gates, we found that a substantial amount of entropy dissipates to the environment (see Fig. 7). This is a fundamental condition for active cooling. Thermodynamically, the control task was carried out very far from equilibrium conditions. In the present study, we did not limit the thermodynamic cost. Such a limit can be achieved by restricting the total energy of the control protocol in the optimization procedure. In turn, this restriction will enable studying the relationship between the thermodynamic cost and the control precision. This could be an interesting future study linking the thermodynamic and complexity resources of the task.

### Algorithmic

The computation efforts required to solve for the optimal control pulse are linked to the complexity of the task. For quantum gates, we expect the resources to scale beyond factorially compared to the state-to-state task (*13*). Finding the control field is equivalent to the job of a quantum compiler, translating an algorithm to a general gate and executing it on the quantum hardware. Because of its complexity, the solution to this problem should be set as a goal for future quantum computers.

In the present study, the chosen algorithm used for optimal control was of the CRAB type (Eq. 12) (*29*). This method uses only initial- and final-state information. Application of control methods that use gradients is anticipated to improve the convergence of the control algorithm. These methods can be implemented with the help of the control equation (Eq. 2) because we have access to the state at transient times. These control methods are expected to be useful for solving more demanding control tasks.

The mathematical issue of controllability underlies the control theory and the required control resources. That is, is a control task theoretically (mathematically) achievable? For a state-to-state transformation of open systems, a controllability criterion was defined in (*10*, *43*). Our state-to-state control tasks comply with these criteria and are therefore controllable. In accordance with the theory (*43*), the CRAB-like random optimization achieved the target state with high fidelity. Similarly, for isolated systems, a controllability criterion has been stated for unitary maps (*24*, *44*). However, a controllability theorem for open-system maps remains an unresolved challenge. Some progress in this direction has been achieved by a recent study that addressed the adiabatic reset problem (*45*) from an optimal control perspective.

The thermodynamics theory provides physical restrictions for the ability to perform a control task. For a setup composed of a system, controller, and thermal environment, thermodynamics imposes a unidirectional flow of energy from the controller through the system to the environment. In addition, a decrease in the system entropy must be accompanied by additional entropy generation in the environment, overall leading to a positive entropy production (see Figs. 1, 3, 5, and 7) (*46*).

A first-principle derivation based on the complete unitary dynamics of the composite system is thermodynamically consistent within the considered validity regime. This property emerges from an initial separable state of the system and environment and the thermally stationary state of the environment (*20*, *47*). A hallmark of thermodynamic consistency is the dependency of the dissipative dynamics on the unitary free dynamics. Previous studies, both experimental and theoretical, have addressed optimal control for cooling transformations under the condition that the unitary (control) and dissipative parts are independent (*48*–*51*). Such an assumption ignores the “dressing” of the system by the field and may violate the laws of thermodynamics (*52*, *53*). By building upon a complete description of the total system (including the field), this discrepancy was fixed and used to achieve control in the present analysis. Within the weak coupling regime, we achieved control objectives unattainable under strict unitary control.

The ability to perform unitary gates under noise resembles ideas from dynamical decoupling (*54*, *55*). The difference is that dynamical decoupling strives to effectively isolate the system from the environment, while the present scheme operates with active heat transport to the environment. The present results serve as a computational demonstration that practical control of gates under dissipative conditions is possible.

To summarize, the presented analysis constitutes a new paradigm for the control of open quantum systems. The theory was demonstrated by studying entropy-changing state-to-state transformations and a universal class of one- and two-qubit unitary gates under external influence.

All explored control protocols were accompanied by substantial entropy production, demonstrating the relevance of thermodynamic principles in the quantum regime. This observation is contrary to the intuitive expectation that unitary controls exist in a decoherence-free subspace (*56*). Notably, for unitary targets, the control trajectory maintains high purity along its path, while the state remains far from the instantaneous attractor, implying large entropy production. This is the hallmark of active cooling.

The obtained protocols can be incorporated into a variety of technological procedures. For example, a standard quantum computation based on the quantum circuit model requires an initial pure state and the ability to perform unitary transformation accurately. In practice, there always exists a classical uncertainty in the initial state because of the finite temperature of the environment. In addition, the idealized quantum gates are subject to external noise, inducing an undesired nonunitary evolution on the qubits. The presented control scheme addresses both problems. Λ_R_ incorporates the environmental influence in the resetting process, allowing to accurately prepare the quantum register in the desired initial state. Moreover, the single-qubit rotation map Λ_U_ and the two-qubit entangling gate $\Lambda_{\sqrt{S}}$ take the dissipation into account. This enables achieving unitary transformations with improved fidelity. These unitary gates constitute a set of universal gates sufficient for the generation of an arbitrary computation (*37*). Using such control in noisy quantum information processing units can potentially boost their performance.

Alternatively, using the present control scheme, one can also induce controlled nonunitary operations. These can be incorporated into the realization of nonunitary quantum computations (*57*, *58*). Last, the ability to generate a directly controlled entropy change can pave the way to previously unidentified cooling (and maybe heating) mechanisms, a research field that has received extensive attention in the past two decades (*6*, *9*–*12*, *35*, *59*).

The capabilities of the model were shown to allow both the generation of nonunitary transformation and an efficient generation of unitary transformation under similar dissipative conditions. This work paves the way for numerous interesting future directions. Promising future directions include the investigation of open-system quantum speed limits, the inclusion of non-Markovian effects, and the embedding of quantum control in the framework of thermodynamics. These, and others, might lead to previously unknown insights that could improve our ability to understand and apply control in the open quantum world.

## METHODS

In this section, we derive the control master Eq. 2 from first principles. This equation of motion served as a working horse for the open-system control.

Consider a driven quantum system interacting with an external environment. The time-dependent Hamiltonian of the composite system is of the form

$$\hat{H}(t)={\hat{H}}_{S}(t)+{\hat{H}}_{E}+{\hat{H}}_{I}$$ (23)

where ${\hat{H}}_{S}(t)$ and ${\hat{H}}_{E}$ are the system and environment Hamiltonians, respectively. The system Hamiltonian is given explicitly in Eq. 4, and the environment is modeled by a bosonic bath with an ohmic spectral density function

$${\hat{H}}_{E}=\sum_{k}(\hbar \omega_{k}{\hat{a}}_{k}^{†}{\hat{a}}_{k}+1/2)$$ (24)

where ${\hat{a}}_{k}$ and ${\hat{a}}_{k}^{†}$ are the annihilation and creation operators of the mode *k*, respectively (designating both the wave vector and the polarization). We consider an interaction term of the following form

$${\hat{H}}_{I}=\hat{S}⊗\hat{B}$$ (25)

where $\hat{S}$ and $\hat{B}$ are Hermitian operators of the system and the environment, respectively. Specifically, we chose $\hat{S}$ to coincide with the angular momentum in the *y* direction, leading to the interaction

$${\hat{H}}_{I}={\hat{J}}_{y}⊗\sum_{k}g_{k}({\hat{a}}_{k}+{\hat{a}}_{k}^{†})$$ (26)

As discussed in Introduction, there are four typical time scales associated with Hamiltonian (Eq. 23): (i) The bare-system time scale τ_S_ can be expressed in terms of the typical Bohr frequencies of ${\hat{H}}_{S}^{0}$: τ_S_ ∼ 1/ω*_S_*. (ii) The environment’s characteristic time scale is associated with the decay of its correlation functions, which can be evaluated by the square inverse of the environment’s spectral width: τ_E_ ∼ 1/Δν. (iii) The relaxation time scale is proportionate to the inverse of the typical coupling strength between the system and environment, τ_R_ ∝ 1/*g*^2^, with *g* ∼ *g_k_*. (iv) The time scale of the driving protocol is associated with the rate of change of the eigenvalues of the free dynamical map, which includes both the bare drift Hamiltonian and the drive. Formally, it is defined as $\tau_{d}∼{\left(\frac{d\theta_{j}}{\mathrm{dt}}\right)}^{-1}$.

Our present goal is to derive an effective equation of motion for the system, influenced by the external degrees of freedom. The typical separation between the four dynamical time scales will serve as the crucial ingredient in the analytical derivation of such a dynamical description.

Assuming weak system-environment coupling and rapid decay of the environmental correlations, τ_S_ ≪ τ_R_ and τ_E_ ≪ τ_S_ allow applying the Born-Markov approximation to the exact dynamical equation of the reduced system in the interaction picture relative to the free dynamics. This leads to the celebrated quantum Markovian master (Eq. 14)

$$\frac{d}{\mathrm{dt}}{\tilde{\rho }}_{S}(t)=-\frac{1}{\hbar^{2}}\int_{0}^{\infty }\mathrm{ds}\;{\mathrm{tr}}_{E}([{\tilde{H}}_{I}(t),[{\tilde{H}}_{I}(t-s),{\tilde{\rho }}_{S}(t)⊗{\hat{\rho }}_{E}]])$$ (27)

In addition, when the environment is sufficiently large, it is only negligibly affected by the interaction with the system, meaning that it remains stationary throughout the dynamics: ${\hat{\rho }}_{E}\equiv {\hat{\rho }}_{E}(0)$. We proceed by expanding the interaction Hamiltonian in terms of the system’s eigenoperators (Eq. 8)

$$\begin{array}{cc} {\tilde{H}}_{I} & ={\hat{U}}^{†}(t){\hat{H}}_{I}\hat{U}(t)\\ & ={\hat{U}}_{S}^{†}(t){\hat{S}\hat{U}}_{S}(t)⊗{\hat{U}}_{E}^{†}(t)\hat{B}\hat{U}(t)\\ & =\sum_{k}c_{k}(t){\hat{U}}_{S}^{†}(t){\hat{F}}_{k}(t){\hat{U}}_{S}(t)⊗\tilde{B}(t)\\ & =\sum_{k}c_{k}(t){\hat{F}}_{k}(t)e^{-i\theta_{k}(t)}⊗\tilde{B}(t)\\ & =\sum_{k}\eta_{k}(t){\hat{F}}_{k}(t)e^{-i\Lambda_{k}(t)}⊗\tilde{B}(t)\\ & =\sum_{k}\eta_{k}(t){\hat{F}}_{k}^{†}(t)e^{i\Lambda_{k}(t)}⊗{\tilde{B}}^{†}(t) \end{array}$$ (28)

where *c_k_* = η*_k_*(*t*)*e*−*i*λ*k*(*t*) is expansion coefficients of $\hat{S}$ in terms of the eigenoperators $\left\{{\hat{F}}_{k}(t)\right\}$, η*_k_*, λ*_k_* ∈ *R*, and Λ*_k_*(*t*) = θ*_k_*(*t*) + λ*_k_*(*t*). In the last equality, we used the Hermiticity of $\hat{S}$ and $\hat{B}$. Next, we substitute Eq. 28 into Eq. 27 to obtain

$$\begin{array}{c} \frac{d}{\mathrm{dt}}{\tilde{\rho }}_{S}(t)=\frac{1}{\hbar^{2}}\int_{0}^{\infty }\mathrm{ds}\;{\mathrm{tr}}_{E}([{\tilde{H}}_{I}(t-s){\tilde{\rho }}_{S}(t){\hat{\rho }}_{E}{\tilde{H}}_{I}(t)-{\tilde{H}}_{I}(t){\tilde{H}}_{I}(t-s){\tilde{\rho }}_{S}(t){\hat{\rho }}_{E}])+h.c\\ =\frac{1}{\hbar^{2}}\sum_{kk^{\prime }}\int_{0}^{\infty }\mathrm{ds}\;e^{-i(\Lambda_{k}(t-s)-\Lambda_{k^{\prime }}(t))}{\langle \tilde{B}(t)\tilde{B}(t-s)\rangle }_{E}\eta_{k^{\prime }}(t)\eta_{k}(t-s)\\ \times [{\hat{F}}_{k}(t-s){\tilde{\rho }}_{S}(t){\hat{F}}_{k^{\prime }}^{†}(t)-{\hat{F}}_{k^{\prime }}^{†}(t){\hat{F}}_{k}(t-s){\tilde{\rho }}_{S}(t)+h.c] \end{array}$$ (29)

Under Markovian dynamics, the environmental correlations decay rapidly relative to the intrinsic time scale of the system. Here, we also assume that the environment dynamics are much faster than the typical time scale of the drive τ_E_ ≪ τ_d_. Under this condition, the integral is dominated by the value of the integrand in the range *s* ∈ [0, τ_E_]. In this physical regime, the eigenoperators and coefficients do not change much, and we can approximate ${\hat{F}}_{k}(t-s)\approx {\hat{F}}_{k}(t)$ and η*_k_*(*t* − *s*) ≈ η*_k_*(*t*), leading to

$$\frac{d}{\mathrm{dt}}{\tilde{\rho }}_{S}(t)=\Xi_{kk^{\prime }}(t)[{\hat{F}}_{k}(t){\tilde{\rho }}_{S}(t){\hat{F}}_{k^{\prime }}^{†}(t)-{\hat{F}}_{k^{\prime }}^{†}(t){\hat{F}}_{k}(t){\tilde{\rho }}_{S}(t)]+h.c$$ (30)

with

$$\Xi_{kk^{\prime }}(t)=\frac{1}{\hbar^{2}}\int_{0}^{\infty }\mathrm{ds}e^{-i(\Lambda_{k}(t-s)-\Lambda_{k^{\prime }}(t))}{\langle \tilde{B}(s)\tilde{B}(0)\rangle }_{E}\eta_{k^{\prime }}(t)\eta_{k}(t)$$ (31)

where we used the invariance of correlation functions under time translation for a stationary environment. The rapid decay of environmental correlations also allows expanding the phases near *t*, as *s* ≃ τ_E_

$$\Lambda_{k}(t-s)=\Lambda_{k}(t)-\Lambda_{k}(t)s\equiv \Lambda_{k}(t)-\omega_{k}(t)s$$ (32)

Substituting Eq. 32 into Eq. 31, we obtain terms proportional to *e*−*i*(Λ*k*(*t*) − Λ*k*′(*t*)). For *k* ≠ *k*′, these typically rotate rapidly and average out to zero. As a result, mixed terms in the master equation vanish, leading to a GKLS form

$$\begin{array}{c} \frac{d}{\mathrm{dt}}{\tilde{\rho }}_{S}(t)=-\frac{i}{\hbar }[{\hat{H}}_{\mathrm{LS}}(\omega_{k}(t),t),{\tilde{\rho }}_{S}(t)]\\ +\sum_{k}\gamma_{k}(\omega_{k}(t),t)({\hat{F}}_{k}(t){\tilde{\rho }}_{S}(t){\hat{F}}_{k}^{†}(t)-\frac{1}{2}\{{\hat{F}}_{k}^{†}(t){\hat{F}}_{k}(t){\tilde{\rho }}_{S}(t)\}) \end{array}$$ (33)

where

$$\gamma (\omega (t),t)=\Gamma (\omega ,t)+\Gamma^{*}(\omega ,t)=\int_{-\infty }^{\infty }\mathrm{ds}\;e^{i\omega s}{\langle \tilde{B}(s){\tilde{B}}_{\beta }(0)\rangle }_{E}$$ (34)

and

$${\hat{H}}_{\mathrm{LS}}(t)=\sum_{k}R(\omega_{k}(t))F_{k}^{†}(t)F_{k}(t)$$ (35)

is the Lamb shift Hamiltonian, with

$$\Gamma (\omega_{k}(t),t)=\frac{1}{\hbar^{2}}\eta_{k}^{2}(t)\int_{0}^{\infty }{\mathrm{dse}}^{i\omega_{k}(t)s}{\langle \hat{B}(s)\hat{B}(0)\rangle }_{E}$$ (36)

and

$$R(\omega ,t)=\frac{1}{2i}(\Gamma (\omega ,t)-\Gamma^{*}(\omega ,t))$$ (37)

Overall, the obtained master equation is valid in the weak coupling limit, assuming a Markovian environment. In addition, the change in the drive may be rapid relative to the system (τ_d_ ∼ τ_S_), but should be slow relative to the decay of environmental correlations. Such a regime allows consistently describing the dynamics of a nonadiabatically driven open quantum system. The rapid drive induces mixing of the system’s energy and coherence, while the environment degrades the coherence and induces energy transfer. The interplay between energy and coherence within the system serves as the prime ingredient in realizing open-system control.

## Supplementary Materials

### This PDF file includes:

Sections S1 and S2 Figs. S1 and S2
