# Supplementary material for: Controlling the uncontrollable: Quantum control of open-system dynamics
Source: Sci Adv. 2022 Nov 2;8(44):eadd0828. doi: 10.1126/sciadv.add0828 (PMC9629718; doi:10.1126/sciadv.add0828)
Supplement: Supplementary file 1 — Sections S1 and S2 Figs. S1 and S2 [file sciadv.add0828_sm.pdf]

Science Advances
AAAS

Supplementary Materials for

Controlling the uncontrollable: Quantum control of open-system dynamics

Shimshon Kallush *et al.*

Corresponding author: Ronnie Kosloff, ronnie@fh.huji.ac.il

Sci. Adv. **8**, eadd0828 (2022)
DOI: 10.1126/sciadv.add0828

This PDF file includes:

Sections S1 and S2
Figs. S1 and S2

S1 Numerical comments

When employing Eq. (8) one needs to retrieve the time-dependent phase of $\hat{F}_j$, the eigenoperators of the propagator $\mathcal{U}_S(t, 0)$. We employ a numerical diagonalization of $\mathcal{U}_S(t, 0)$ at each time step. We then ensure a continuous labeling of the eigenoperators. This task is achieved by calculating the overlap between the current and updated set of operators. A conventional retrieval of the phase with inverse trigonometric functions was found to suffer from erratic behaviour. In order overcome this issue we employed the following procedure: For an unknown phase function $f(t) = e^{i\theta(t)}$, the formal derivative of the function is given by:

$$\frac{d}{dt}f(t) = i\dot{\theta}f \rightarrow \theta = i\int_0^t \frac{\dot{f}dt}{f} \tag{S1}$$

so that the phase is retrieved stably by integration.

Another important numerical comment concerns the number of frequency components M, in Eq. (12). The number of frequencies required to modify the entropy in a cooling and heating

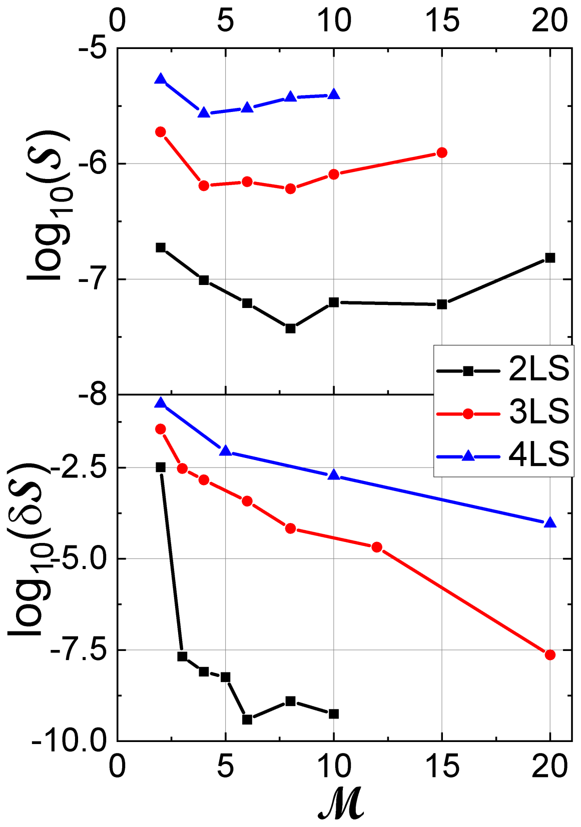

Figure S1: The entropy difference as the objective functional as a function of the number of field frequency components M . The control task is compared for two (black), three (red) and four (blue) level systems, for cooling (upper panel) and heating (lower panel) processes.

protocol are displayed in Fig. S1. Typically, the objective improves with the number of frequencies in the control. However, in the cooling fields, we witness a saturation with the increase of M. In addition, due to the increase in complexity achieving the objective becomes increasingly harder when increasing the number of energy levels.

S2 Scaling of the objective with the system environment coupling

The deviation from the control objective increases with the system-environment coupling g, Eq. (11). Figure S2 shows a log-log plot of the degradation of the transformation as a function of the effective decay rate $\Gamma = k_{\downarrow} + k_{\uparrow}$, which is calculated in the absence of driving.

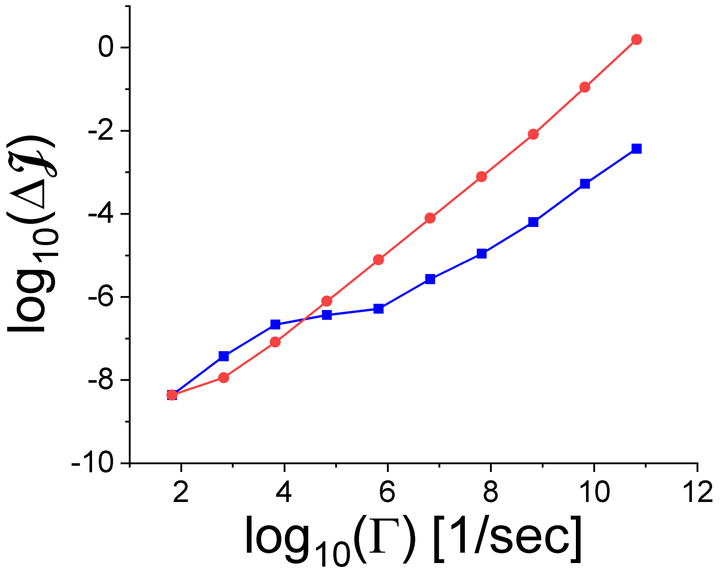

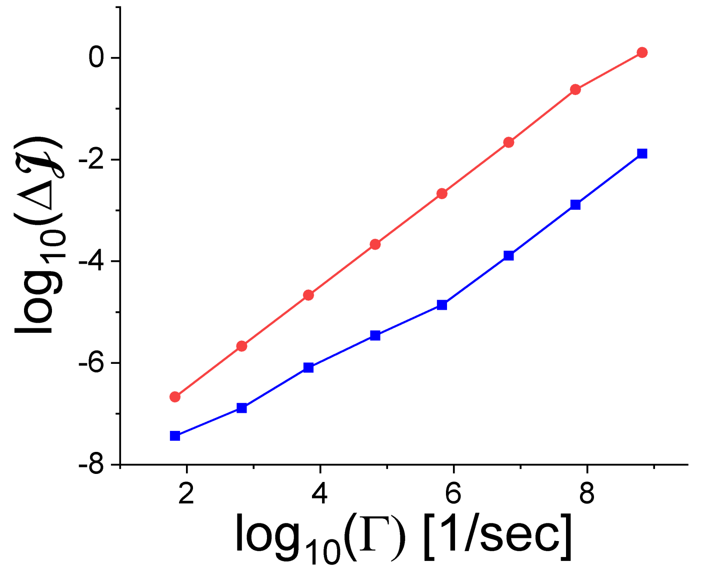

Figure S2: Degradation of a Unitary transformation: The precision $\Delta\mathcal{J}$ in log scale as a function of decay parameter $\Gamma = k_{\downarrow} + k_{\uparrow}$ for the drift Hamiltonian ($\hat{V}(t) = 0$). To Hadamar transformation, bottom two qubit gate. (blue) The infidelity for the optimal transformation as a function of system-environment coupling. Each point represents the best case optimization computed from scratch for a given coupling strength. (red) The infidelity obtain form the bath free optimization when the bath is included. Cf. Fig. 6.
